# Transient Receptor Potential (TRP) Channels in Head-and-Neck Squamous Cell Carcinomas: Diagnostic, Prognostic, and Therapeutic Potentials

**DOI:** 10.3390/ijms21176374

**Published:** 2020-09-02

**Authors:** Fruzsina Kiss, Krisztina Pohóczky, Arpad Szállási, Zsuzsanna Helyes

**Affiliations:** 1 Somogy County Kaposi Mór Teaching Hospital, H-7400 Kaposvár, Hungary; k.fruzsina17@gmail.com; 2 Department of Pharmacology, Faculty of Pharmacy, University of Pécs, H-7624 Pécs, Hungary; 3 Department of Pharmacology and Pharmacotherapy, Medical School, University of Pécs, H-7624 Pécs, Hungary; zsuzsanna.helyes@aok.pte.hu; 4 János Szentágothai Research Centre, Centre for Neuroscience, University of Pécs, H-7624 Pécs, Hungary; 5 1st Department of Pathology and Experimental Cancer Research, Semmelweis University, H-1085 Budapest, Hungary; szallasi.arpad@med.semmelweis-univ.hu; 6 PharmInVivo Ltd., H-7629 Pécs, Hungary

**Keywords:** head-and-neck squamous cell carcinoma, TRP channels, calcium influx, malignant transformation, diagnostic and prognostic biomarker, therapeutic target

## Abstract

Head-and-neck squamous cell carcinomas (HNSCC) remain a leading cause of cancer morbidity and mortality worldwide. This is a largely preventable disease with smoking, alcohol abuse, and human papilloma virus (HPV) being the main risk factors. Yet, many patients are diagnosed with advanced disease, and no survival improvement has been seen for oral SCC in the past decade. Clearly, new diagnostic and prognostic markers are needed for early diagnosis and to guide therapy. Gene expression studies implied the involvement of transient receptor potential (TRP) channels in the pathogenesis of HNSCC. TRPs are expressed in normal epithelium where they play a key role in proliferation and differentiation. There is increasing evidence that the expression of TRP channels may change in HNSCC with important implications for diagnosis, prognosis, and therapy. In this review, we propose that TRP channel expression may afford a novel opportunity for early diagnosis of HNSCC and targeted molecular treatment.

## 1. Introduction

With approximately 650,000 new cases reported worldwide annually, head-and-neck cancers remain a leading cause of cancer mortality [1]. Despite recent advances in therapeutic options, approximately 40% of the newly diagnosed patients will die of their disease [1]. More than 90% of head-and-neck cancers are squamous cell carcinomas (HNSCC) [2]. Historical risk factors for HNSCC include tobacco use and alcohol consumption [3]. In fact, the decreasing incidence of HNSCC in developed countries coincides with the decline in smoking habits. By contrast, in high-risk countries like India and Pakistan (where bidi smoking and betel quid chewing are rampant), HNSCC is the most common cancer in men [4]. In our home country, Hungary, HNSCC still has high prevalence (the average annual number of HNSCC patients is 13,600 in an insured population of 9.5 million) and imposes significant economic burden [5].

The epidemiology and risk factors of HNSCC are changing. In developed countries, “never-smokers, never-drinkers” now represent an emerging, unique clinical subgroup of young patients [6]. The etiological role of high-risk human papilloma virus (HPV) infection in HNSCC is also widely recognized thanks to high-profile patients. In fact, in the US, approximately one in every five new HNSCC cases can at present be attributed to HPV [7].

There is an increasing recognition that HNSCC represents a heterogenous group of biologically distinct cancers with complex molecular abnormalities [8]. For example, by gene expression microarray of 375 genes, two distinct patient groups could be distinguished: one group of mostly young patients with poor prognosis, and another group of predominantly older patients with good prognosis [9]. Another study detected at least 4 distinct molecular subtypes of HNSCC [10]. These gene expression assays are useful for research purposes, but they are not yet suited for routine use. Furthermore, there is a lack of agreement among pathologists in the recognition of premalignant conditions, in the grading of dysplasias and separating non-progressing dysplasias from those progressing into invasive cancer [11]. A better understanding of the molecular underpinnings of HNSCC has the promise of developing novel tools to diagnose, prognosticate, and treat the disease.

Many proteins in cancer cells show altered (increased or decreased) expression compared to normal cells, and this may be used as an ancillary diagnostic tool and/or to guide therapy. For example, estrogen receptor (ER) is expressed in the endometrium, but not in the endocervix, thus ER-immunoreactivity can distinguish between endometrial and endocervical adenocarcinoma [12]. Furthermore, in endometrial (and also breast) carcinoma, ER-positivity correlates positively with prognosis and predicts good response to hormonal (e.g., tamoxifen) treatment [13]. Clearly, an ER-like marker in HNSCC would be of great value for diagnosticians and clinicians.

Transient Receptor Potential (TRP) channels are expressed in normal epithelial cells where they are thought to play an important role in proliferation and differentiation [14,15]. Moreover, TRP channels are expressed in cells (neurons, endothelium, and immune cells) that form the cancer microenviroment [16]. There is a growing body of evidence that the expression of TRP channels can change during malignant transformation [17,18,19]. Indeed, several lines of experimental evidence suggest an oncogenic role for TRP channels in various cancers like melanoma, glioblastoma, prostate, or breast adenocarcinoma [20,21,22]. By comparison, little is known about the role of TRP channels in HNSCC. Since distinct cancers share similar molecular pathways, one can argue that TRP channel findings in other cancers also apply (at least to a certain degree) to HNSCC.

In this review, we critically evaluate the evidence that altered TRP channel expression in HNSCC may be used as a diagnostic aid to recognize malignant transformation and/or progression to invasive disease. We also examine the literature data if altered TRP channel expression may help to identify a group of HNSCC patients with poor prognosis who could benefit from more aggressive therapy. Last, we ask the question if drugs targeting TRP channels may kill tumor cells. This has practical importance, because the oral cavity is particularly amenable to topical anticancer therapy targeting TRP channels.

## 2. TRP Channels: From Fruit Flies to Human Cancer

In 1969, a fruit fly eye mutant called “trp” (transient receptor potential) was described that responded to lasting light stimulation with a transient depolarizing after-potential instead of the normal prolonged response [23]. The wild type *trp* gene was isolated and shown to rescue this phenotype. That is, the wild type *trp* channel causes a persistent (and not transient) current: in other words, “trp channel” is really a misnomer. The peculiarities of this naming convention aside, the biological behavior of TRP channels is in contrast to many ion channels, which fully adapt when exposed to constant stimulation [24].

As of today, the mammalian TRP channel superfamily has 28 members (27 in humans) [25]. Based on sequence homology, the family has been divided into six subfamilies: canonical (TRPC1 to C7), vanilloid (TRPV1 to V6), melastatin (TRPM1 to M8), ankyrin (TRPA1), mucolipin (TRPML1 to ML3), and polycystin (TRPP1 to P3) [26]. Although these proteins share some structural similarities, there are enough differences to develop subtype-selective compounds [27]. Of note, genetic defects in TRPs (so-called “TRP channelopathies”) are increasingly recognized causes of hereditary human disease [28]. In a much simplified manner, TRPs are multifunctional signaling molecules, expressed in a wide variety of tissues and cell types [26]. Some TRPs function as non-selective cation channels in the plasma membrane, whereas others regulate Ca^2+^ release in intracellular organelles [27]. Most TRPs are polymodal channels (so-called “coincidence detectors”) that are activated by both physical (temperature, voltage, pressure, and tension) and chemical stimuli [27].

In the context of oral physiology, it is noteworthy that TRPV1 is the receptor for volatilized acids, capsaicin, and piperine (the pungent ingredients in red and black pepper, respectively) [29,30], TRPA1 responds to garlic, cinnamon, horseradish, wasabi, and methyl-salicylate [29,30,31]. TRPM8 is a molecular target for menthol (which, in high concentrations, also can activate TRPA1) [32].

TRP channels have been among the most aggressively pursued drug targets over the past few years. The therapeutic potential of TRP channel modulators for pain, respiratory disease, etc., are detailed elsewhere [24,25,26,27]. Here, it suffices to mention that a growing body of evidence implicates TRP channels in oncogenesis [17,18,19]. Indeed, the founding member of the TRPM (melastatin) subfamily, TRPM1, was discovered by comparing *benign nevi* and malignant melanoma [33,34,35]. Of note, the expression of the *TRPM1* gene is inversely correlated with aggressiveness of the cancer, implying a tumor suppressor gene [36].

## 3. TRP Channel Expression in Normal Epithelial Cells

Keratinocytes express a variety of TRP channels, which are thought to be involved in key functions like growth, differentiation, survival, and inflammation [37]. In 1997, the laboratory of David Julius was the first to identify the rat capsaicin (vanilloid) receptor via an expression cloning strategy that took advantage of the Ca^2+^ conductance [38]. The human isoform showed largely similar properties [39]. Within the TRP superfamily, the capsaicin receptor as TRPV1 is the founding member of the now populous TRPV (vanilloid) subdivision, from TRPV1 to TRPV6. The crystal structure of the TRPV1 protein was largely solved by cryoelectron microscopy, including a chemically more native lipid nanodisc environment [40]. The *Trpv1* knockout mouse (which misses exon 13 that codes mainly the pore loop and transmembrane domain 6) looked normal, but lacked responses to capsaicin and exhibited minimal inflammatory thermal hyperalgesia [40,41]. Of note, the *Trpv1* knockout mouse showed no distinct skin phenotype.

Capsaicin sensitivity was long considered a hallmark of primary sensory neurons [42,43]. Therefore, it took the field by surprise that TRPV1 was found to be expressed, albeit at much lower levels than in sensory neurons, in non-neuronal cell types of the human skin, airways, and bladder [43]. TRPV1 has been implicated in cutaneous growth and differentiation [44]. One can speculate that epithelial TRPV1 (skin to TRPV1 in sensory neurons) may function as a sensor of harmful environmental stimuli. Indeed, TRPV1 is activated by chemicals (both acids and alkalines) that can damage the cornea, and TRPV1 activation was shown to facilitate wound repair after cornea injury [45]. Similarly, in the rat gingiva activation of TRPV1 by capsaicin stimulated epithelial cell proliferation [46]. However, in cultured keratinocytes, TRPV1 activation was reported to suppress proliferation and promote apoptosis which would impair the repair of damaged tissue [47].

The effect of TRPV1 activation on hair growth is not less confusing, with some studies reporting increased hair growth [48], whereas others finding the opposite outcome following topical capsaicin administration [49]. Finally, TRPV1 antagonists (AMG-980 and SB-705498) had no effect on keratinocyte proliferation [50]. Clearly, the physiological role epithelial TRPV1 remains to be elucidated.

TRPV3 [51] and TRPV4 [10] are also expressed in epidermal keratinocytes. In mice, epidermal TRPV3 is crucial in promoting barrier formation and hair morphogenesis. Indeed, the *Trpv3* knockout mouse has a characteristic wavy hair coat [52], whereas the constitutively active gain-of-function mutation *Trpv3^Gly573Ser^* results in a hairless phenotype [53]. Moreover, the skin-targeted *Trpv3^Gly573Ser^* transgenic animals spontaneously develop an inflammatory condition associated with scratching behavior similarly to human atopic dermatitis [54]. TRPV3 is expressed in all cell layers of the human epidermis [55]. It is of special relevance to this review that TRPV3 expression in the oral mucosa is higher than in the skin keratinocytes [53].

TRPV4 is a warm temperature sensor in human keratinocytes, as well as a critical component in the molecular machinery that responds to UV-B light [56,57]. Recently, a gain-of-function mutation in *TRPM4* has been linked to erythrokeratoderma [58]. In the rat, the oral epithelium was found to express all members of the TRPV receptor subfamily (with the exception of *Trpv5*) along with the *Trpm2* and *Trpm8* genes, with regional differences among the buccal, palatal, and tongue mucosa [59]. The oral epithelial cells showed elevated intracellular Ca^2+^ levels after capsaicin (TRPV1), camphor (with limited selectivity for TRPV3), 4-α-phorbol 12,13 didecanoate (TRPV4), 2-aminoethoxydiphenylborate (TRPV2), and menthol (that preferentially activates TRPM8) challenge, implying the existence of functional TRP channels [59,60].

Moving downstream from the oral cavity, TRPV4 expression was described in both primary human airway epithelial cells and cell lines (e.g., A549, Beas 2B, and NCI-H292) [61]. In NCI-H292 cells, selective TRPV4 agonists trigger Ca^2+^ influx and increase the release of interleukin-8 (IL-8) and prostaglandin E (PGE) [61]. In bronchial epithelial cells, TRPV1 controls the expression of the pro-inflammatory cytokines, IL-6 and IL-8 [62]. TRPA1 is expressed in cultured human airway epithelial cells, smooth muscle cells, and fibroblasts [63]. TRPM8 is also expressed in human lung epithelial cells, where its activation increases the expression of several cytokines and chemokines [64].

## 4. TRP Channels in Cancer Cells: A Role in Tumorigenesis?

Cancer progression is associated with the suppression of pathways leading to cell death (apoptosis) and shifting the balance towards uncontrolled cell proliferation. Ca^2+^ signaling plays a key role in these cellular mechanisms, implicating an important role of Ca^2+^ permeable ion channels like TRPs in tumorigenesis [62,65,66,67,68].

TRP channels can increase the intracellular Ca^2+^ concentration either by acting as Ca^2+^ entry pathways in the plasma membrane or by releasing Ca^2+^ from internal stores such as the mitochondria and the endoplasmic reticulum [68]. This altered Ca^2+^ signal may change gene transcription, cell proliferation, differentiation, migration, invasion, motility, and apoptosis via calcium-dependent signaling cascades such as the mitogen-activated protein kinase/extracellular signal regulated kinase (MAPK/ERK) and PI3K/Akt pathways. Ca^2+^ entering the tumor cell can activate calmodulin, initiating the activation of extracellular signal regulated kinases (ERK). In turn, ERK can activate several regulatory targets in the cytoplasm, including the MAPK-activated protein kinases ribosomal s6 kinase, MAPK interacting protein kinase and mitogen and stress activated protein kinase, and the protease calpain. These proteins have important regulatory roles in cell cycle progression, cell survival, and nuclear signaling.

**Figure 1:**
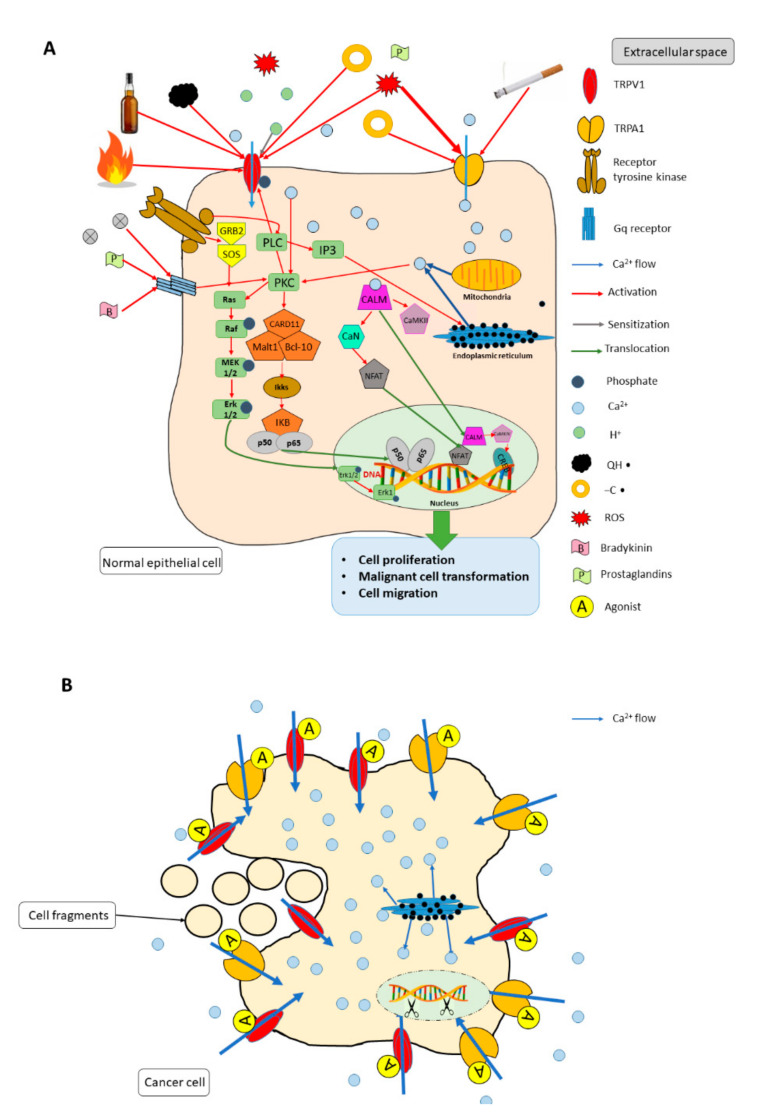
The complex role of transient receptor potential vanilloid 1 (TRPV1) and ankyrin 1 (TRPA1) ion channels in normal epithelial cell (**A**) and cancer cell (**B**). Panel A represents the TRPV1-, TRPA1-mediated signal transduction pathways of potentially carcinogenic exogenous compounds as well as endogenous ligands. Panel B illustrates the antitumor effect of TRPV1, TRPA1 receptor agonists on a cancer cell. Abbreviations: TRPV1: transient receptor potential vanilloid 1; TRPA1: transient receptor potential ankyrin 1; QH^•^: hydroxyl radical; -C^•^: carbon-centered radical; ROS: reactive oxygen species; GRB2: growth factor receptor-bound protein 2; MEK1/2: mitogen-activated protein kinase kinase; ERK: extracellular signal regulated kinase; PLC: phospholipase C; PKC: protein kinase C; CARD11: caspase recruitment domain-containing protein 11; Malt1: mucosa-associated lymphoid tissue lymphoma translocation protein 1; Bcl-10: B-cell lymphoma/leukemia 10; Ikss: IKK alpha + IKK beta; IKB: nuclear factor of kappa light polypeptide gene enhancer in B-cells inhibitor alpha; IP3: inositol trisphosphate; CALM: calmodulin; CaN: carbonic anhydrase 1; NFAT: nuclear factor of activated T-cells; CREB: cAMP-response element binding protein.

Activated ERK also translocates to the nucleus where it influences gene transcription and translation patterns by phosphorylating the transcription factors, signal transducer and activator of transcription 3 (STAT3), and nuclear factor of activated T-cells (NFAT) [69]. On the other hand, the ERK pathway has a pro-apoptotic role in particular cell lines: that is, Ca^2+^ influx through TRP channels can either positively or negatively regulate apoptosis through activation of the ERK pathway (Figure 1A) [70].

Most TRP channels exhibit cation-selectivity with preference to divalent cations (Ca^2+^ and Mg^2+^) [26]. TRPs can be activated or sensitized directly by ligand binding or indirectly via downstream intracellular signaling pathways through other receptors [68,69]. TRP ligands include exogenous small molecules (e.g., capsaicin for TRPV1, allyl isothiocyanate and allicin for TRPA1, icilin for TRPM8), endogenous lipids (e.g., diacyl-glicerol for TRPC3 and TRPC6), eicosanoids (e.g., prostaglandins for TRPV1 and TRPV4), purine nucleotides, protons (e.g., H^+^ for TRPV1), ions (e.g., Ca^2+^, Mg^2+^), and by the Ca^2+^/calmodulin complex (e.g., TRPV1 and TRPA1). Direct activation of the TRPs can also occur by temperature changes, voltage, mechanical stimulation, and reactive oxidative species (ROS) [70,71,72]. Indeed, TRPM2, TRPC5, TRPV1, and TRPA1 are ROS-sensitive Ca^2+^-permeable channels, with TRPA1 showing the greatest sensitivity [73,74]. Oxidation of disulfide bonds on the pore-forming region and cytoplasmic N-terminal region of TRP channels is essential for the channel activation, while reduction of thiols to disulfides may inhibit the channel [75]. All these endogenous ligands are produced in a higher concentration by cancer cells and other cell types (e.g., immune cells, vascular endothelial cells, and fibroblasts) that form the microenvironment of the tumor.

Smoking is a well-known risk factor in HNSCC with special emphasis on oral cavity and laryngeal cancer. Cigarette smoke contains 15 known carcinogenic agents, including *N*-nitrosamines, polycyclic aromatic hydrocarbons (PAHs), and aromatic amines [76]. Although PAHs are biologically inert materials, with the help of metabolic activation, they are converted to harmful secondary metabolites, such as radical-cation intermediates and ROS [77]. Cigarette smoke and tar also have high concentrations of stable free radicals, identified as a hydroxyl radical (QH^•^), and carbon-centered radicals (-C^•^). Tar extracts in aqueous buffers (such as saliva) and other solvents can reduce oxygen to form superoxide, and superoxide can dismutate to form hydrogen peroxide [78]. Cigarette smoke, however, does not cause mutations in genes encoding TRP channels, but instead alters the expression levels of wild-type channels at the mRNA and/or protein levels. This can also affect the gating and/or activation properties of the channel (Figure 1A) [79].

TRPV1 is the best studied TRP channel in cancer. The evidence linking TRPV1 to carcinogenesis, however, remains controversial and speculative [80]. In a 26-week dermal oncogenicity study, topical capsaicin (the prototypical TRPV1 agonist) did not increase papilloma formation in the mouse skin treated by the tumor promoter, 12-*O*-tetradecanoylphorbol-13-acetate (TPA) [81]. The ultrapotent capsaicin analog, resiniferatoxin, did not promote tumor formation either [82]. Furthermore, the TRPV1 antagonists AMG-980 and SB-705498 had no impact on skin carcinogenesis [50]. In addition, mice with pharmacologically inactivated (desensitized by resiniferatoxin) TRPV1 showed no increase in papilloma growth in the two-stage skin carcinogenesis model (Blumberg and Szallasi, unpublished observations). These negative results are in conflict with the postulated tumor suppressor role of TRPV1 in melanoma [83] and colon cancer [84]. Importantly, so far, there is no report of increased skin cancer incidence in the human skin following high-dose capsaicin patch (Qutenza) treatment [85].

## 5. Epigenetic Regulation of TRP Channel Expression: A Role in Malignant Cell Transformation?

Epigenetic regulation is an inheritable cellular change which is not encoded in the DNA sequence. Broadly speaking, it can be divided into three main categories: (1) DNA methylation, (2) histone modification, and (3) genomic imprinting [86]. Epigenetic changes are crucial during key physiological processes such as cell proliferation, differentiation, and morphogenesis. In the last decade, a wide range of epigenetic mechanisms were found to be altered in different types of cancers, and their manipulation is a promising new area for cancer prevention, detection, and therapy [87,88,89,90].

Certain regions of the TRP receptor coding sequences are targets for epigenetic modification. For example, elevated histone H3 acetylation of the *Trpv1* promoter region leads to increased expression of TRPV1 in dorsal root ganglia and promotes hyperalgesia in rats [91]. Moreover, SUMOylation (covalent modification by small ubiquitin-related modifier polypeptide) was recently shown to play an important role in TRPV1 regulation during diabetes, protecting the channel from metabolic damage and exogenous stressors [92].

*TRPM2* promoter can be hypomethylated after hydrogen-peroxide stress, which induce channel overexpression, abnormal Ca^2+^ influx, and mitochondrial damages. The methylation of human *TRPA1* promoter region results in elevated TRPA1 expression and altered pain perception [93]. In erytroleukemic cells, transactivation of the *TRPA1* promoter via Notch1 receptor intracellular domain induces TRPA1 expression and suppresses erythroid differentiation. In breast cancer cells, chemoresistance is mediated by miR-320a by targeting TRPC5 and NFATc3 [17].

In HPV-positive HNSCC patients, the methylation profile of certain genes involved in signal transduction (e.g., EGFR-ERK and PI3-K/AKT pathways) was found to be altered compared to HPV-negative patients, implying an important role for epigenetic regulation in the oncogenesis [94]. However, the epigenetic regulation of TRP receptors in HNSCC, and its role, if any, in malignant transformation is yet to be determined.

## 6. TRP Channel Expression in the Tumor Microenvironment

A number of TRP channels (including TRPA1, TRPV1, TRPV4, TRPM3, TRPM4, TRPM8, TRPC3, and TRPC6) are expressed in immune cells (lymphocytes and macrophages), sensory nerve endings, vascular endothelial and smooth muscle cells, and stromal cells in the vicinity of tumors [16,19]. These channels can influence the tumor microenvironment by modulating sensory–vascular–immune–tumor interactions [26]. Activation of the sensory and non-neuronal channels by a variety of mediators produced by the tumor can exert a complex feed-back on carcinogenesis. The tumor microenvironment is a promising therapeutic target.

Neoangiogenesis is crucial for tumor growth. TRP channels, such as TRPC3, TRPC6, TRPV1, TRPV4, and TRPM4, can regulate vascular permeability and angiogenesis. There is compelling evidence that angiogenic growth factors such as vascular endothelial growth factor (VEGF) can activate/sensitize TRP channels, resulting in increased intracellular Ca^2+^ concentration and angioneogenesis [65]. This is important, because VEGF inhibitors like bevacizumab are already in clinical use in the management of various cancers (breast, colon, renal cell, non-small cell lung, etc.). VEGF inhibitors are, however, plagued by side-effects like hypotension, blood clots, and impaired surgical wound healing [83,84,87,88]. It remains to be seen if TRP channel modulators can block neoangiogenesis without the adverse effects of existing VEGF inhibitors [95,96].

Tumor-related formation of new vessels is initiated in a hypoxic environment principally due to the secretion of growth factors that can sensitize a variety of TRP channels via tyrosine-kinase-linked receptors. This is supported by experimental finding obtained in the in vivo matrigel plug angiogenesis assay where the highly selective TRPV1 receptor agonist, evodiamine, induced angiogenesis in the wild-type, but not in the *Trpv1* knock out mouse [97].

## 7. TRP Channels as Potential Diagnostic and Prognostic Tools in Cancer

### 7.1. TRPV Subfamily

In the rat, TRPV1, TRPV2, TRPV3, and TRPV4 mRNAs are detectable in healthy oral mucosa, with greater expression in the tongue and oral floor than the gingiva and bucca. The expression of TRPV1 and TRPV3 is higher than that of TRPV2 and TRPV4. TRPV1-, TRPV2-, and TRPV3-like immunoreactivity is seen predominantly in the basal layer of the epidermis, whereas TRPV4 shows diffuse positivity [98]. In the healthy human tongue, TRPV1-like immunoreactivity is confined to the basal layer where it is sparse and weak [99]. Human larynx epithelium expresses only TRPV1 and TRPV4 [100].

TRPV1 expression is significantly upregulated in human HNSCC in all layers of the epidermis compared to the normal mucosa (where it is present in the basal layer only) in several locations such as tongue, oral floor, gingiva, and bucca [98]. Furthermore, TRPV1-like immunopositivity was detected in the human tongue HNSCC cell lines, SCC4, C25, and HSC3. The TRPV1 mRNA expression in HSC3 cells is relatively similar to that in normal keratinocytes, but its relative expression is significantly higher in SCC4 and SCC25 cells [101]. Importantly, TRPV1 is increased in leukoplakia compared to healthy controls [99]. Taken together, these findings suggest that increased TRPV1-like immunoreactivity may aid pathologists in distinguishing reactive and malignant oral squamous mucosal lesions. Interestingly, the reverse seems to be true for kidney cancer where TRPV1 is strongly expressed in normal renal tubules with much reduced (or absent) expression in renal cell carcinoma [102]. Moreover, TRPV1 is a potential negative prognostic marker in breast cancer where TRPV1 expression in the endoplasmic reticulum and Golgi apparatus (and/or the surrounding of these structures) heralds poor prognosis [103]. It remains to be determined if this observation also holds true for HNSCC (Table 1).

TRPV2 expression has been studied in various cancers (but not in HNSCC). TRPV2 is a potential biomarker and therapeutic target in hepatocellular carcinoma [112]. The TRPV2 agonist, probenecid, inhibited the growth of HepG2 xenografts in SCID mice [113]. Increased TRPV2 expression predicted higher disease-free survival is a subset of patients with triple-negative breast cancer [114]. TRPV2 overexpression is also present in patients with advanced esophageal squamous cell carcinoma associated with metastatic disease [104]. In prostatic adenocarcinoma, TRPV2 overexpression predicts the castration-resistant phenotype. In invasive urothelial carcinoma, TRPV2 expression positively correlates with the histologic grading [115]. By contrast, TRPV2 is reduced in *glioblastoma multiforme* (GBM) compared to normal astrocytes [116]. It would be interesting to see if TRPV2 carries prognostic value in HNSCC.

TRPV4 is highly expressed in all layers of the healthy epidermis. TRPV4 protein and mRNA levels are, however, significantly downregulated in both premalignant lesions (e.g., actinic keratosis) and malignant non-melanoma skin cancers compared to healthy human skin. This suggests that epigenetic or other factors act similarly in both precancerous and malignant skin phenotypes (Table 2) [117].

TRPV6 is overexpressed in advanced prostate cancer with little or no expression in healthy and benign prostate tissue [120]. In breast cancer, TRPV6 is up-regulated, compared to adjacent, benign breast tissue. Furthermore, TRPV6 positively correlated with the grade of cancer: the higher the grade, the higher the TRPV6 expression. The intensity of TRPV6 staining was higher in invasive than in in situ carcinoma. TRPV6 silencing induced significant decrease in TRPV6 mRNA in both non-invasive (MCF-7) and invasive (MDA-MB-231) breast cancer cell lines [121,122]. By contrast, in esophageal squamous cell carcinoma down-regulated TRPV6 was associated with unfavorable 3-year disease-specific survival (Table 1, Table 2) [118].

### 7.2. TRPM Subfamily

The founding member of the TRPM subfamily, TRPM1, was discovered in 1998 as a protein present in benign nevi, but not in malignant melanoma. It was speculated that TRPM1 is a tumor suppressor, hence the name “melastatin” [28,29].

Human tongue SCC samples (22 out of 23) show significantly increased TRPM2 mRNA and protein levels as detected by both immunohistochemistry (IHC) and Western blots (WB). Enhanced TRPM2 expression was also detected in the nuclei of SCC cells. The function of TRPM2 may depend on its membrane or nuclear locations. Of note, TRPM2 mRNA is also up-regulated in the non-cancerous tongue mucosa of SCC patients, consistently with the concept of field cancerization. The activation of the TRPM2 channel by H_2_O_2_ increases apoptosis of SCC cells through the p53 and p21 pathways. TRPM2 (and its long noncoding RNA *TRPM2-AS*) is also overexpressed in prostate cancer [107].

TRPM3 promotes growth and autophagy in renal cell carcinoma, TRPM4 is elevated in diffuse large B-cell lymphoma, whereas TRPM5 is believed to play an oncogenic role in melanoma [123,124,125].

TRPM7 overexpression was found in 102 out of 206 nasopharyngeal carcinoma (NPC) tissues. Expression levels positively correlate with lymphatic involvement and distant metastasis, predicting shorter survival time. Elevated TRPM7 mRNA was detected in some metastatic NPC samples compared to primary NPC samples. By contrast, in normal nasopharyngeal mucosa, only few TRPM7 mRNA copies were detected. Taken together, these findings indicate that TRPM7 may be a valuable prognostic marker in NPC patients [107].

Similar to the nasopharynx, TRPM7 is undetectable in the normal esophageal epithelium, while it is expressed in the cytoplasm of esophageal SCC cells. In the esophageal SCC cell lines, TE5 and KYSE70, increased TRPM7 mRNA levels were detected with RT-PCR [109]. Functional TRPM7 is observed in breast cancer cell lines (MCF-7 and hBCE cells) with a role in cell proliferation [126]. The proliferative role of TRPM7 has been demonstrated in other tumors including pancreatic adenocarcinoma, breast carcinoma, T-cell leukemia, rat basophilic leukemia, retinoblastoma, and glioblastoma [127].

TRPM8 is expressed in prostate cancer with negative correlation to tumor severity: as the cancer progresses, its TRPM8 expression decreases [128]. Similar to prostate, in the breast, TRPM8 expression is found mainly in well-differentiated invasive ductal carcinoma [129].

### 7.3. TRPA Subfamily

TRPA1-like immunopositivity is upregulated in human NPC samples, with negative predictive value for disease-specific, metastasis-, and local recurrence-free survival. If confirmed in independent studies, TRPA1-like immunoreactivity could be a valuable prognostic marker in HNSCC patients treated by irradiation and adjuvant cisplatin chemotherapy [105].

### 7.4. TRPC Subfamily

TRPC3, TRPC4, and TRPC6 expression was described in the human breast carcinoma cell lines, TD7, MCF-7, and MDA-MB-231 [130,131,132] (Table 1, Table 2). In breast cancer, the level of TRPC1 and TRPC6 expression was similar in the noninvasive and invasive areas, regardless of the ER status. Although the expression of TRPC6 in hBDA primary cell cultures was high, no correlation was found between the expression and the tumor stage, histological type, and/or the lymph node metastasis [121].

### 7.5. TRPP2 Subfamily

TRPP2 protein expression is significantly increased in human laryngeal carcinoma as shown by WB and IHC, with negatively correlation to the survival time of the patients. Knocking down TRPP2 in the human laryngeal carcinoma cell line, Hep2, suppressed ATP-induced Ca^2+^ release, migration, invasion, and the epithelial-mesenchymal transition process (Table 1) [111].

## 8. TRP Channels as Therapeutic Targets in Cancer

In theory, Ca^2+^-permeable TRP channels in cancer cells are attractive therapeutic targets, since their activation causes high intracytoplasmic Ca^2+^ levels that, in turn, could activate the apoptotic pathways. In practice, it is problematic to achieve sufficiently high concentrations of the TRP agonists in most tumors without causing intolerable side effects. For example, high TRPV1 expression was reported in *glioblastome multiforme*, implicating TRPV1 as a therapeutic target. However, one can only wonder how to deliver high capsaicin (or resiniferatoxin) doses to the brain without killing the patient. HNSCC represents a unique opportunity to kill tumor cells with topical TRP channel agonist (or antagonist) administration.

TRPV1 is an interesting target to explore in HNSCC for two reasons. One, TRPV1 expression confined to the basal layer in normal epithelium, is increased in all layers of HNSCC. Two, potent TRPV1 agonists (e.g., capsaicin and resiniferatoxin) are readily available for the studies. Experiments were performed, but unfortunately, the results are conflicting.

In TRPV1-null mice, increased papilloma incidence and multiplicity were reported. Based on this observation, it was speculated that TRPV1 functions as a tumor suppressor: it blocks skin carcinogenesis via down-regulating epidermal growth factor receptor (EGFR) expression by Cbl (ubiquitylation enzyme)-mediated EGFR ubiquitination, and subsequently its degradation through the lysosomal pathway. In other words, the absence of TRPV1 will lead to increased total EGFR protein with enhanced activation of downstream signaling components of the EGFR pathway [131]. If so, how can TRPV1 expression be increased in HNSCC? In addition, why do we not see increased papilloma formation in mice whose TRPV1 were ablated by resiniferatoxin pretreatment? Obviously, the most important experiment is yet to be done: can topical capsaicin be curative in HNSCC patients? Or, can we treat skin SCC with a high-concentration capsaicin patch like Qutenza? Of note, intrathecal resiniferatoxin ameliorated cancer pain in dogs with osteosarcoma, but did not slow down the progression of the tumor.

TRPA1 is another intriguing target to explore. Similarly to TRPV1, it is highly expressed in HNSCC cells [105], and potent TRPA1 agonists are readily available for the studies (Figure 1B).

Other potential therapeutic targets include TRPV3, TRPV6, and TRPM2, and TRPM7.

## 9. Discussion and Future Research Directions

There is good evidence that the expression level of several TRP channels like TRPV1 and TRPA1 exhibits significant differences between cancerous and normal tissues [17,18,19]. In principle, these channels can be used as diagnostic markers of malignancy. Other TRP channels seem to have prognostic value. For example, TRPV2 overexpression predicts adverse outcome in patients with esophageal SCC [103], and TRPM7-positive NPC patients also have worse prognosis than those with TRPM7-negative cancer [126]. As of today, it is unclear if altered TRP channel expression is the cause or consequence of the disease. Unfortunately, changes in TRP expressions appear to be cancer-specific. For instance, TRPV1 expression is increased in HNSCC [3] and prostate cancer [132] but is markedly reduced or absent in renal cell carcinoma [102] or urothelial carcinoma [116]. Moreover, TRPV2 overexpression heralds poor prognosis in esophageal SCC [105], but identifies a subgroup of triple-negative breast cancer patients with favorable prognosis [114]. Therefore, observations done in one type of cancer cannot be extrapolated to a different type of cancer.

TRPM2 is a particularly intriguing channel in that its expression is increased not only in HNSCC, but also in the apparently non-cancerous mucosa of these patients [106]. This raises the possibility that with a TRPM2 activator we can eliminate the area of field cancerization and thereby prevent development of secondary malignancies.

In conclusion, TRP channels are promising diagnostic markers, putative prognostication aids, and speculative therapeutic targets in HNSCC. The initial results are promising, but the lion’s share of work still needs to be done.

## Figures and Tables

**Table 1:** Increased expression of Transient Receptor Potential (TRP) channels in human head-and-neck cancer samples and experimental data.

Ion Channel	Methods/ Experimental Techniques/Cell Lines	Results	Cancer Type	Reference
TRPV1	- IHC; WB; RT-qPCR - human tissue samples	protein and mRNA upregulation	OSCC and leukoplakia	[99]
TRPV1	- IHC; RT-qPCR - SCC4, SCC25, and HSC3 OSCC cell lines	protein upregulation and in SCC4, SCC25 cell lines mRNA upregulation	OSCC	[101]
TRPV2	- IHC - human tissue samples	cytoplasmic expression in carcinoma cells widely varied (no, weak, and strong) and strong expression is an independent poor prognostic factor	ESCC	[104]
	- TE5, TE8, TE9, TE15, KYSE70, LYSE150, and KYSE170 ESCC cell lines - RT-qPCR	mRNA is strongly upregulated in TE15, and more strongly in KYSE170 cell lines than in the other ESCC cell lines. Similar intensity of TRPV2 protein expression is observed in TE5, TE9, TE15, KYSE70, and KYSE170.		
	-WB	TRPV2 expression in TE15 and KYSE170 was similar in Western blotting		
TRPV1-4	- IHC; RT-qPCR - human tissue samples	protein and mRNA upregulation in OSCC	OSCC	[98]
TRPA1	-IHC - human tissue samples	upregulation is independently and negatively predictive disease-specific, distal metastasis-free, and local recurrence-free survivals in NPC	NPC	[105]
TRPM2	-IHC - human tissue samples	protein upregulation in tongue SCC	OSCC	[106]
	SCC-9 and SCC-25 OSCC cell lines	regulation of migration and survival of HSCC cells		
TRPM7	-IHC+WB -RT- qPCR - human tissue samples	cytoplasmic membrane and cytoplasmic staining in NPC tissues protein upregulation in 102 out of 206 NPC samples mRNA upregulation insome metastatic NPC samples compared to primary NPC samples and few TRPM7 is found in normal NP tissues	NPC	[107]
	5-8F, 6-10B, CNE1, CNE2, SUNE1, C666-1, HNE1, HONE1, NP16, and NP69	expression of TRPM7 is higher in cells of the CNE2 line, which are capable of metastasizing		
TRPM7	-IHC -human tissue samples	protein is expressed in the cytoplasm of carcinoma cells	ESCC	[108]
	-TE2, TE5, TE9 TE13, KYSE70, and KYSE170	mRNA upregulation is observed in the TE5 and KYSE70 cell lines		
TRPM7	SUNE1 5-8F 6-10B	migration progress of cancer in NPC cell lines 5-8F and 6-10B metastasis	NPC	[109]
TRPM7	FaDu and SCC25 cells	increases proliferation	HNSCC	[72]
TRPM8	HSC3 and HSC4 OSCC cell lines	mRNA upregulation protein on plasma membrane and IC region cell invasion	OSCC	[110]
TRPP2	-WB+IHC - human tissue samples	protein upregulation in human laryngeal carcinoma, with negatively correlation to the survival time of the patients	laryngeal SCC	[111]
	Hep2 cell human laryngeal cell line	Knocking down suppresses ATP-induced Ca2+ release, migration, invasion, and the EMT process		

OSCC: oral squamous cell carcinoma, NPC: nasopharyngeal carcinoma, ESCC: esophageal squamous cell carcinoma IHC: immunohistochemistry, WB: Western blot, qPCR: quantitative polymerase chain reaction, RT-qPCR: real-time quantitative PCR, SK: solar keratosis, BD: Bowen’s disease BCC: basal cell carcinoma, ESCC: esophageal squamous cell carcinoma, HNSCC: head-and-neck squamous cell line, HNMSC: human non-melanotic skin cancer, EMT: epithelial-mesenchymal transition.

**Table 2:** Decreased expression of TRP channels in human head-and-neck cancer samples and experimental.

Ion Channel	Methods/Experimental Model	Results	Cancer Type	Reference
TRPV4	-IHC+WB -RT- qPCR - human tissue samples	downregulation in keratocytes in HNMSC	SK, BD, invasive cutaneous SCC and BCC	[117]
TRPV6	-IHC -RT- qPCR - human tissue samples	-mRNA downregulation is detected in 32 of 45 ESCC tumors -protein downregulation is detected in 118 of 244 ESCC tumors	ESCC	[118]
	KYSE30, KYSE140, KYSE180, KYSE 410, KYSE510, KYSE520, HKESC1, CE81T, EC109, and EC9706	mRNA down-regulation		
TRPC1	CNE2	motility and invasive abilities	NPC	[119]

## Abbreviations

BCC	Basal cell carcinoma
BD	Bowen’s disease
-C•	Carbon-centered radical
EMT	Epithelial-mesenchymal transition
ER	Estrogen receptor
ERK	Extracellular signal regulated kinase
ESCC	Eosophageal squamous cell carcinoma
HNMSC	Human non-melanotic skin cancer
HNSCC	Head-and-neck squamous cell carcinoma
HPV	Human papilloma virus
IHC	Immunohistochemistry
IL	Interleukin
MAPK	Mitogen-activated protein kinase
NFAT	Nuclear factor of activated T-cells
NPC	Nasopharyngeal carcinoma
PAHs	Polycyclic aromatic hydrocarbons
PGE	Prostaglandin E
PI3K	Phosphoinositide 3-kinase
QH^•^:	Hydroxyl radical
qPCR	Quantitative polymerase chain reaction
RT-qPCR	Real-time quantitative PCR
ROS	Reactive oxygen species
SK	Solar keratosis
STAT3	Signal transducer and activator of transcription 3
TPA	12-*O*-tetradecanoylphorbol-13-acetate
TRP	Transient receptor potential
TRPA	Transient receptor potential ankyrin
TRPC	Transient receptor potential canonical
TRPM	Transient receptor potential melastatin
TRPML	Transient receptor potential mucolipin
TRPP	Transient receptor potential polycystin
TRPV	Transient receptor potential vanilloid
VEGF	Vascular endothelial growth factor
WB	Western blot
